# Molecular Mechanisms of Herbicide Resistance in Weeds

**DOI:** 10.3390/genes13112025

**Published:** 2022-11-03

**Authors:** Joel Torra, Ricardo Alcántara-de la Cruz

**Affiliations:** 1 Agrotecnio Centre for Research, Universitat de Lleida, Avda. Rovira Roure 191, 25198 Lleida, Spain; 2 Centro de Ciências da Natureza, Universidade Federal de São Carlos—Campus Lagoa do Sino, Buri 18290-000, Brazil

Herbicides have become one of the most widespread weed-control tools in the world since their advent in the mid-20th century [1]. Nowadays, they are still being used in most conventional cropping systems in modern agriculture [2]. Unfortunately, the persistent use of herbicides is being threatened by the spread of herbicide resistance, a fast evolutionary process that took place a few years after their arrival into modern agriculture [2,3]. To safeguard their future use in agriculture, there is great interest in understanding the molecular mechanisms conferring resistance or predisposing weeds toward evolving herbicide resistance.

Herbicide resistance is governed by target-site resistance (TSR) and non-target-site resistance (NTSR) mechanisms [4]. TSR-based resistance is caused by any gene alteration able to change the interaction with the encoded target protein/enzyme so that the herbicide is not able to sufficiently interfere with it to cause plant death. TSR mechanisms are usually better understood because there is a single well-known target gene, and therefore, they are monogenic [5]. On the other hand, NTSR mechanisms are those not involving the target protein and can decrease the herbicide arriving at the site of action (SoA) into an insufficient amount, so plants can survive; more rarely, any mechanism protecting plants from herbicide damage is also referred as NTSR [5]. NTSR mechanisms are rarely fully understood since they can be quantitative in nature and controlled by several genes (with each gene providing some level of resistance); in other words, NTSR-based resistance can be polygenic [4].

The increase in multiple herbicide resistance to different SoAs, mainly through enhanced metabolism, is of great concern [2]. Multiple herbicide resistance reflects an evolutionary process by which populations or plants can accumulate different resistance mechanisms (TSR and/or NTSR), conferring resistance to several SoAs [6]. Sadly, this process usually occurs because resistance to one SoA provokes switching to another SoA rather than reducing herbicide-selection pressure [7]. Among NTSR mechanisms, enhanced metabolism is the most threatening because, as a generalist mechanism, it can confer cross-resistance to dissimilar herbicide chemistries, even to those never used before [4]. Conversely, TSR is governed by specialist mechanisms, always specific to a single SoA [5].

This Special Issue was focused on the new well-characterized cases of herbicide resistance, both for TSR and/or NTSR (if a molecular basis is reported), as well as studies that identify new gene alterations conferring TSR or the genetic basis involved in NTSR. Both TSR and NTSR can also be divided into different mechanisms depending on their nature. Point mutations, altered expression, or codon deletion of the target-site gene are among the most reported types of TSR mechanisms [5]. NTSR mechanisms usually involve altered patterns of herbicide absorption, translocation, or metabolism. Herbicide-metabolism-based resistances are complex and often involve genes that are members of large gene families, including cytochromes P450 (P450) and Glutathione-S-transferases (GST) [4]. Therefore, this editorial focuses on the nature of the resistance mechanisms of the two major types, TSR and NTSR, described in each of the contributions to this Special Issue.

## 1. Target-Site Resistance Mechanisms

Three articles reported mechanisms of TSR nature covering most of the known types: point mutations [8,9], codon deletion [10], and altered expression patterns [9].

### 1.1. Point Mutations

Resistance to ACCase- and ALS-inhibiting herbicides conferred by point mutations in the gene were among the first cases reported as TSR decades ago. Two contributions reported point mutations conferring resistance to ACCase and ALS inhibitors in *Lolium multiflorum* [8] and *Echinochloa* ssp. [9], respectively. Surprisingly, there are still recent discoveries regarding ACCase-inhibiting herbicides. Kaudun et al. [8] found a novel mutation in position 2027 of the *ACCase* gene, leading to the amino acid change Tryptophan by Leucine. This W2027L change, together with three well-characterized I1781L, I2041T, and D2078G changes also found in the study, endowed resistance to aryloxyphenoxypropionates, phenylpyraxolines, and cyclohexanediones, as well as unidentified NTSR.

Regarding ALS-inhibiting herbicides, point mutations involved in resistance in two *Echinochloa* species from Italian rice were identified [9]. The two species carried different point mutations previously described as conferring resistance: A122N in *E. crus-galli* and W574L in *E. oryzicola*.

### 1.2. Codon Deletion

Resistance to protoporphyrinogen oxidase (PPO) inhibitors in *Amaranthus palmeri* is mainly contributed by the deletion of glycine210 codon (ΔG210), which renders herbicide molecules ineffective [11]. A contribution to this Special Issue functionally validated this codon deletion in other plant species, showing that when ΔG210-PPO was overexpressed in rice and *Arabidopsis thaliana*, it conferred tolerance to fomesafen applied pre-emergence and cross-tolerance to saflufenacil in *A. thaliana* [10]. Moreover, the expression of *A. palmeri* ΔG210-PPO2 also conferred tolerance to fomesafen in rice and *A. thaliana* when soil-applied, together with cross-tolerance to saflufenacil in Arabidopsis [10]. This tolerance trait to PPO inhibitors could be introduced into crops for weed management and herbicide resistance alleviation.

### 1.3. Altered Expression Patterns

TSR can be due to increased expression of the target-site gene as well, producing more protein than can be inhibited by field herbicide doses. Gene overexpression can be due to increased numbers of genomic copies and/or regulatory changes increasing transcription [5]. Panozzo et al. [9] described the mechanisms involved in ALS-inhibiting herbicides in *E. crus-galli* and *E. oryzicola*. Though point mutations of the *ALS* gene were the main TSR mechanism conferring resistance, apparently, an alteration of the expression patterns also rendered resistance. When the relative expression of the different *ALS* gene copies was evaluated, *ALS1* was significantly more expressed than the other copies (*ALS2* and *ALS3*) in both species. Interestingly, mutations were only carried by the overexpressed gene copy *ALS1*. This study indicates that high resistance levels to ALS inhibitors in *Echinochloa* ssp. can not only be conferred by point mutations but also by the overexpression of gene copies carrying them [9].

## 2. Non-Target-Site Resistance Mechanisms

Among NTSR mechanisms, most articles cover an enhanced metabolism involving different enzyme families and degradation pathways. One contribution referred to P450 [12] and two to GST [12,13], the most important enzyme families involved in herbicide degradation, while two other contributions highlighted that stress-responsive genes were involved in herbicide metabolism [12,14].

### 2.1. Cytochrome P450

In the study by Chandra and León [12], four gene groups encoding putative NTSR enzymes, namely, P450, GST, uridine 5′-diphospho-glucuronosyltransferase (UDPGT), and nitronate monooxygenase (NMO), were analyzed using monocot and dicot gene sequences from publicly available databases. Phylogenetic trees revealed that most of the P450-resistance-related sequences belonged to CYP81. P450 has been associated with resistance to ACCase, ALS, and microtubule-assembly-inhibiting herbicides in some grass weed species [15,16]. Further, the 3D structure of the CYP81 enzyme was predicted, and a proline-rich membrane pivot was identified as the substrate-recognition site using a homology approach [12].

### 2.2. Glutathione-S-Transferase

After P450, the second-most important protein family involved in NTSR mechanisms are GST, phase-II enzymes that can conjugate herbicides with the tripeptide glutathione [5]. According to phylogenetic trees using public databases for monocots and dicots, among different GST classes, tau first and phi second were those most related to herbicide resistance [12]. Previous studies have shown that both GST classes can confer resistance to different herbicide SoAs [17,18]. Finally, the G-site and the H-site of the GST were identified as the binding sites for the herbicide-resistant enzymes.

In another study by González-Torralva and Norsworthy [13], the resistance mechanism in a trifluralin-resistant *A. palmeri* population from the USA was investigated. Results indicated that applying a GST inhibitor showed significant differences in root length between susceptible and resistant plants, indicating that GSTs are probably involved in the resistance mechanism. On the other hand, no point mutations or differences in the gene copy number were found, revealing that GST-mediated metabolism can contribute to trifluralin (a microtubule assembly inhibitor) resistance in *A. palmeri*.

### 2.3. Other NTSR Mechanisms

The detailed SoA of quinclorac, a synthetic auxin with herbicide activity against grass weeds, is not fully understood, and the same is true for the potential resistance mechanisms evolving in weeds. A contribution to this Special Issue tried to unravel the resistance mechanisms of *E. colona* to this herbicide using a de novo transcriptome approach [14]. Data showed that carbon metabolism, photosynthesis, and ureide metabolism, all indicating improved metabolic efficiency, were upregulated in the resistant population. Interestingly, following quinclorac application, trehalose biosynthesis and many antioxidant defense elements were also upregulated, which are known for abiotic stress mitigation.

Similarly, the contribution by Chandra and León [12] provided data suggesting that stress-responsive pathways in plants can also be involved in herbicide resistance. The study of promoter sequences of NTSR genes revealed stress-related motifs, as well as eight transcription factor binding sites (TFBS). The discovered TFBS and the identified motifs play key roles in mitigating abiotic stress.

## 3. Summary

Contributions in this Special Issue reported on herbicide resistance in several grass weed species and one broadleaf weed. Among grasses, *Echinochloa* ssp. is reported in two articles [9,14] and *L. multiflorum* in one [8]; among dicots, two articles reported on the global invasive weed species *A. palmeri* [10,13]. All these weeds are acknowledged to be among the top 10 weed species resistant to most SoAs globally [19]. *A. palmeri* is already the third-ranking global species in terms of its resistance to more SoAs, *E. crus-galli* is fourth, *L. multiflorum* is sixth, and *E. colona* is 11th [19].

Resistance to five SoAs, auxin mimics, ALS, ACCase, PPO, and microtubule-assembly-inhibiting herbicides, is reported in this Special Issue, which agrees with the SoA herbicides most related to herbicide resistance worldwide [19]. Resistance to post-emergence herbicides appears in three articles, while two refer to pre-emergence herbicides in different cropping systems. Interestingly, information on the resistance to auxins in *Echinochloa* ssp. [14], microtubule assembly [13], and PPO inhibitors [10], both in *A. palmeri*, are contributions of this Special Issue.
